# No difference in patella mobility between conventional mechanically aligned and robotic‐assisted functionally aligned total knee arthroplasty

**DOI:** 10.1002/ksa.70380

**Published:** 2026-04-09

**Authors:** Krzysztof Lachowski, Karim Mustapha Naja, Robert Prill, Andreas Niemeier, Mikhail Salzmann, Roland Becker

**Affiliations:** ^1^ Center of Orthopaedics and Traumatology University Hospital Brandenburg/Havel, Brandenburg Medical School Theodor Fontane Brandenburg an der Havel Germany; ^2^ Faculty of Health Science Brandenburg Medical School Theodor Fontane Potsdam Germany; ^3^ Department of Orthopaedics and Traumatology Hospital Reinbeck St. Adolf‐Stift GmbH Reinbeck Germany; ^4^ Department of Orthopaedics University Medical Center Hamburg‐Eppendorf Hamburg Germany

**Keywords:** outcome, patella mobility, robotic‐assisted surgery, total knee arthroplasty

## Abstract

**Purpose:**

Robotic‐assisted (RA) surgery allows for higher accuracy in total knee arthroplasty (TKA) and for better standardization of alignment concepts. It was hypothesized that the more personalized component placement in functional robotic‐assisted total knee arthroplasty (RA‐TKA) would show an impact on patella mobility and clinical outcome.

**Methods:**

Prospective study was performed on 80 patients who received TKA (Triathlon; Stryker). In 51 patients, the prosthesis was implanted using MAKO robotic assistance (RA‐TKA), while 29 patients underwent conventional implantation (conventional total knee arthroplasty [C‐TKA]). Patella mobility was compared pre‐ and postoperatively after three months. Medial and lateral translation was measured in extension by using a patellostabilometer (PSM), which measures medio‐lateral patella mobility under a force of 10 N. Clinical assessment was based on Oxford Knee Score (OKS), Knee Society Score (KSS), Kujala and Western Ontario and McMaster Universities Arthritis Index (WOMAC) scores.

**Results:**

The medio‐lateral patella mobility was 31 ± 9 mm before and 34 ± 7 mm (*p* < 0.01) after surgery. There was no significant difference in mediolateral patella mobility between the two groups (*p* = 0.282). Patella mobility increased by 3 mm in both groups after surgery. The medial and lateral shift ranged between 14 and 18 mm. All clinical scores (OKS, KSS, Kujala and WOMAC score) improved significantly from before to after surgery (*p* ≤ 0.001) without difference between the groups.

**Conclusions:**

RA‐TKA did not result in changes in patella mobility when compared to conventional implantation. Other factors, such as general joint laxity or implant design and component placement, may have a more significant impact on patella mobility and clinical outcome.

**Level of Evidence:**

Level II prospective designed study.

**Abbreviations**
AKP: anterior knee pain
ASA‐Score: American Society of Anesthesiologists physical status classification system
BMI: body mass index
FJS: Forgotten Joint Score
ICC: intraclass correlation
KSS: Knee Society Score
OA: osteoarthritis
OKS: Oxford Knee Score
PROM: patient‐reported outcome measures
PSM: patellostabilometer
ROM: range of motion
TKA: total knee arthroplasty
WOMAC: Western Ontario and McMaster Universities Arthritis Index

## INTRODUCTION

Patella mobility plays an important role in total knee arthroplasty (TKA). Decreased patella mobility causes anterior knee pain (AKP), reduced knee range of motion and inferior function [7, 17, 21]. Patella tracking depends on femoral component placement in both the coronal and axial planes and is of utmost importance for the functional outcome. The patellofemoral compartment is regarded as an independent third compartment of the knee [3, 18]. Correct component placement is challenging because the natural morphology of the patellofemoral compartment shows high variability [23].

Previous studies have shown that femoral and tibial malrotation have an impact on patella tracking [6]. By changing patellar tracking, peripatellar soft tissue structures may come under increased tension, which in turn can cause activation of nociceptors at the medial and lateral retinaculum. An increase in lateral patella tilt results in lower scores for both the Knee Society Score (KSS) and the Western Ontario and McMaster Universities Osteoarthritis Index (WOMAC) [6]. Nociceptors are mainly located in the joint capsule, which makes the capsule one of the most sensitive structures of the knee [11]. Thus, a reduction in patella mobility due to an increase in soft tissue tension may be a reason for AKP and knee stiffness [5, 27].

Robotic‐assisted total knee arthroplasty (RA‐TKA) represents a significant technological advancement in orthopaedic surgery, allowing enhanced precision in component alignment and soft tissue balancing [9]. Image‐based systems using preoperative computed tomography (CT) scans improve surgical planning for a well‐matched femoral and tibial component placement close to the natural anatomy of the knee. The surgeon plans optimal implant position, bone covering and limb alignment based on a preoperative virtual model. The robot helps to execute the planning with high accuracy, but does not propose any component position.

It is important to note that there is a high degree of interindividual variability in the orientation of the trochlea groove [14]. It has been shown recently that more personalized alignment in TKA better restores the patella groove than a mechanical alignment, namely, conventional TKA (C‐TKA) [24]. More personalization in component placement, such as in the concept of functional alignment, may therefore also replicate physiological soft‐tissue tension around the patella better than conventional mechanical alignment. It is conceivable that following the anatomy of the knee more closely could improve peripatellar soft tissue tension.

The impact of RA‐functional alignment on patella mobility and clinical outcome remains unclear. It was hypothesized that the more personalized component placement in functional RA‐TKA may influence patella mobility and clinical outcome.

## METHODS

This prospective study was conducted at the University of Brandenburg an der Havel. Group RA‐TKA included patients who received RA‐TKA using the MAKO robotic platform (Version 2.0) (*n* = 51) and Group C‐TKA were operated in a conventional fashion using the standard manual surgical technique for mechanical alignment (*n* = 29). Patients in the RA‐TKA group were operated on using a functional alignment approach, starting with restoration of the patients' joint line obliquity within 5° of varus and adjusting femoral component placement by taking lower limb alignment and gapping into account. The study cohort included 47 females and 33 males with a mean age of 73.9 ± 9.1 years. Osteoarthritis of Grade IV according to Kellgren and Lawrence was confirmed radiologically, and all patients had remained clinically symptomatic after three to six months of conservative treatment prior to surgery. Inclusion criteria were patients undergoing primary TKA using the CR‐design of Triathlon® implant by Stryker without patella resurfacing. RA surgery was performed routinely; however, some patients had conventional surgery for logistical reasons, which were unrelated to patients' demographics or co‐morbidities. Exclusion criteria were previous open surgeries at the knee, generalized ligament laxity, neuromuscular disease or signs of inflammation.

Mediolateral patella mobility was measured in extension using the patellostabilometer (PSM) for medial and lateral patella movement starting from the neutral position [19, 20]. The knee was stabilized in the PSM by a pad placed against the medial and lateral epicondyles to stabilize the knee, and the patella was moved in both directions by the manipulator applying a force of 10 N. Displacement was recorded continuously during testing and finally stored in a specifically designed database. The instrument was validated recently showing an absolute deviation of all measurements of 1.5 mm [19]. Patella mobility was measured before surgery and three months after surgery.

Clinical assessment was based on the Oxford Knee Score (OKS), KSS, Kujala‐Score, Forgotten Joint Score (FJS) and McMaster Universities Osteoarthritis Index (WOMAC) recorded prior to and 3 months after surgery.

Sample size was based on previous study results with the aim of finding a medium effect, respecting the chance of 2.3 mm measurement error and using a predefined allocation ratio of 0.7.

The study was approved by the ethical committee of the Medizinische Hochschule Brandenburg, No. E‐02‐20200405. The participants were informed about the purpose of the study and signed informed consent.

The primary outcome was postoperative patellar mobility in both directions related to baseline measurements prior to surgery. Statistics were performed using SPSS V.30.1. Data were analysed using descriptive statistics, Pearson correlation and Spearman correlation coefficients as well as dependent *t* test. A *p* value < 0.05 was considered statistically significant. In case of significant differences, Cohen's *d* effect size measures are shown.

## RESULTS

The total medio‐lateral patella mobility of the study cohort was 31 ± 9 mm before and 34 ± 7 mm after surgery. The medial shift of the cohort was 15 ± 6 mm and the lateral shift 16 ± 4 mm prior to surgery and 17 ± 4 mm and 17 ± 5 mm after surgery. The medio‐lateral patella mobility is shown for both groups separately in Table 1.

**Table 1:** Medial and lateral patella shift before and after surgery of the RA‐TKA group and the C‐TKA group without statistical significance.

	Medial shift—pre	Medial shift—post	Lateral shift—pre	Lateral shift—post
RA‐TKA	15 ± 4 mm	16 ± 4 mm	14 ± 5 mm	16 ± 4 mm
C‐TKA	15 ± 4 mm	17 ± 5 mm	15 ± 6 mm	17 ± 4 mm

Abbreviations: C‐TKA, conventional total knee arthroplasty; RA‐TKA, robotic‐assisted total knee arthroplasty.

The difference in the overall patella mobility before and after surgery was analysed for both groups. There was no statistically significant difference before and after surgery in either group, and no difference between the groups (*p* = 0.424) (Figure 1).

**Figure 1:**
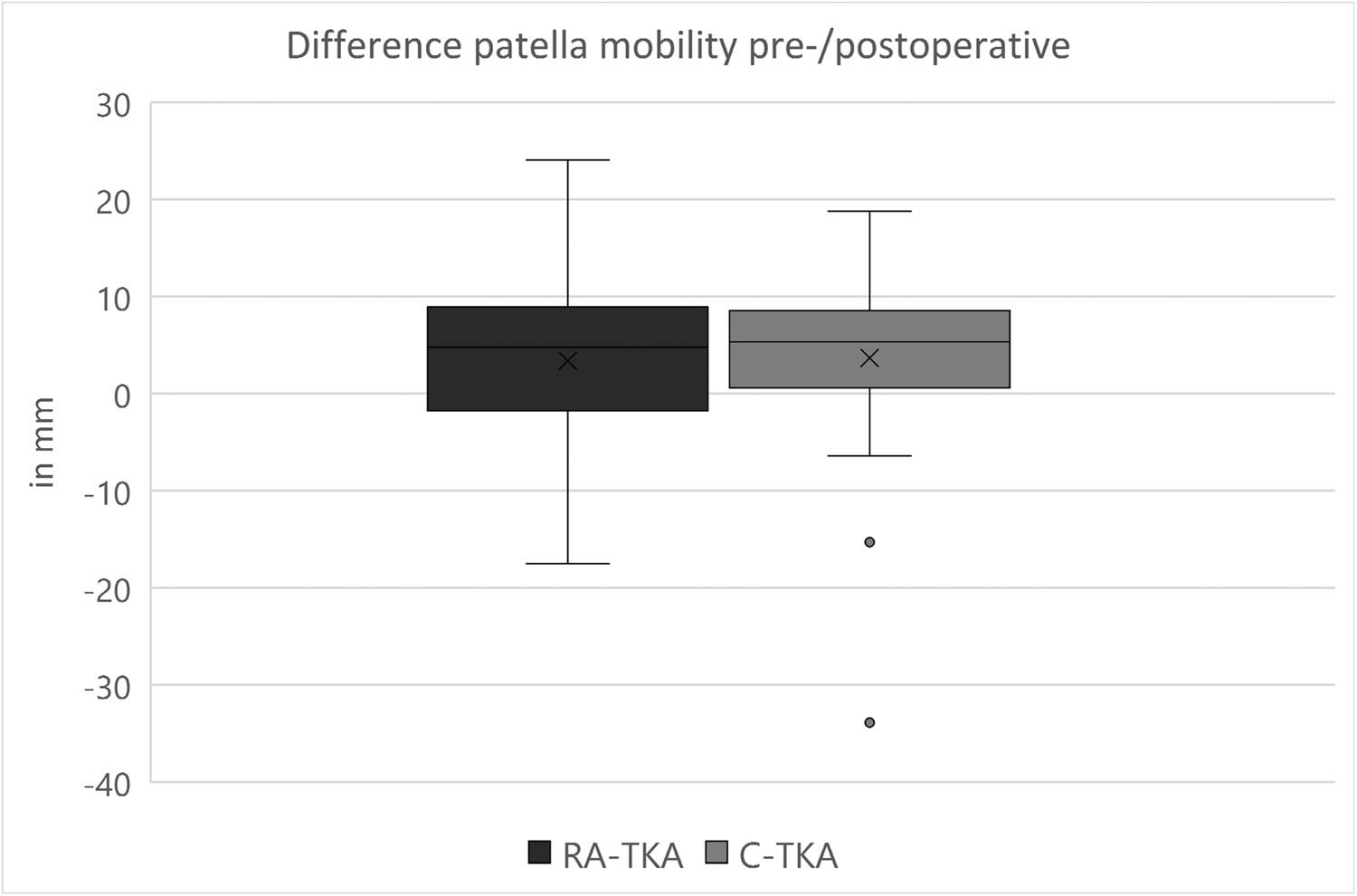
Difference of patella mobility before and after surgery in mm in the RA‐TKA and C‐TKA group. C‐TKA, conventional total knee arthroplasty; RA‐TKA, robotic‐assisted total knee arthroplasty.

A highly significant improvement in all scores was found between the pre‐ and postoperative assessment (Table 2).

**Table 2:** Clinical outcome before and after surgery based on OKS, Kujala, KSS, FJS and WOMAC.

Score	Mean	Standard error	Paired *t* test *p* value
OKS—pre	20.8 ± 6.8	0.76	<0.001
OKS—post	37.2 ± 7.8	0.87	
Kujala—pre	44.4 ± 12.8	1.44	<0.001
Kujala—post	74.6 ± 15.3	1.73	
KSS—pre	68.54 ± 23.4	2.62	<0.001
KSS—post	129.1 ± 26.5	2.97	
FJS—pre	47.6 ± 16.2	1.81	<0.001
FJS—post	34.6 ± 13.2	1.48	
WOMAC—pre	89.7 ± 41.6	4.66	<0.001
WOMAC—post	186.3 ± 46.8	5.24	

Abbreviations: FJS, Forgotten Joint Score; KSS, Knee Society Score; OKS, Oxford Knee Score; WOMAC, Western Ontario and McMaster Universities Arthritis Index.

There was no difference between the two groups in clinical improvement from before to after surgery (Table 3).

**Table 3:** No difference between the group RA‐TKA and C‐TKA in terms of clinical outcome based on OKS, KSS, Kujala, FJS and WOMAC prior and after surgery.

	RA‐TKA	C‐TKA	*p* value
OKS—pre	20.4 ± 6.4	21.5 ± 7.3	n.s.
OKS—post	36.2 ± 8	39 ± 5.3	n.s.
KSS—pre	68.4 ± 21.2	68.7 ± 27.2	n.s.
KSS—post	126.3 ± 29.2	134.1 ± 20.5	n.s.
Kujala—pre	45 ± 11.4	43.2 ± 14.7	n.s.
Kujala—post	70.3 ± 16.1	82.1 ± 10.5	n.s.
FJS—pre	53.8 ± 9.8	36.8 ± 19.5	n.s.
FJS—post	34.6 ± 14.3	34.6 ± 11.2	n.s.
WOMAC—pre	83.8 ± 32.7	100.1 ± 52.3	n.s.
WOMAC—post	180.4 ± 52.8	196.9 ± 32.2	n.s.

Abbreviations: C‐TKA, conventional total knee arthroplasty; FJS, Forgotten Joint Score; KSS, Knee Society Score; n.s., not significant; OKS, Oxford Knee Score; RA‐TKA, robotic‐assisted total knee arthroplasty; WOMAC, Western Ontario and McMaster Universities Arthritis Index.

We found no clinically meaningful correlation between the change of patella mobility and the change of clinical parameters (*r* = −0.03 to 0.13).

## DISCUSSION

The most important finding of the present study was no significant difference in patella mobility between RA functional and conventional mechanical alignment TKA using a single implant design. These findings suggest that while robotic assistance may enhance precision in bony preparation and component placement, it does not necessarily translate into improved soft tissue tension of the medial and lateral retinaculum or enhanced knee function. Improved accuracy in execution of the preoperative plan and the more personalized approach in functionally aligned TKA do not seem to have an impact on patella mobility.

There are several potential explanations for our findings. First, the design of the chosen implant may already support patella kinematics so well that the potential gain associated with RA surgery over manual mechanical alignment may become very small in relation and thus not measurable anymore. Specific features of the implant include a wide and deepened trochlea groove and a 7° orientation of the anterior flange of the femoral component to avoid patellofemoral overstuffing at the early stage of knee flexion. The trochlea groove is oriented at a 6° angle, allowing the patella to enter the groove and move medially during knee flexion [16]. The design of the Triathlon implant emphasizes the asymmetric and more anatomic trochlear groove more than other TKA implants on the market. Although in theory, these are design features that should contribute to a well‐aligned postoperative patellar tracking, previous studies have reported that there was no difference in patella tracking between a symmetrical and asymmetrical femoral component design [28]. In addition, even with the more anatomical design such as used here, a relevant mismatch persists between the natural trochlea and the implant trochlea groove. Therefore, it is questionable to what extent it may be desirable or clinically important to aim for an exact anatomical restoration of the trochlear groove in TKA [2, 10].

Second, the placement of the femoral component may influence the orientation of the trochlear groove to a higher degree than the implant design itself. The trochlea groove tends to run more medially when aligned functionally and kinematically aligned than in the mechanically aligned position. Therefore, the trochlea groove may require some adaptation when employing modern alignment philosophies [15]. In the present study, patella mobility was measured solely in extension, where the patella enters the trochlea groove. Thus, the current data do not allow conclusions about the patella mobility throughout the entire range of knee motion.

The entrance point of the trochlea groove is important, as significant changes may occur in both the coronal and transversal planes during component placement. Varus‐valgus orientation in extension and internal external rotation in flexion affect the trochlear groove's orientation, depending on component placement [4]. Previous anthropometric studies have reported significant variations between Whitside's line and the MPTA. New studies suggest that the third patellofemoral compartment should be considered as an independent one and not directly related to the medial and lateral femorotibial compartments [17].

These aspects were taken into consideration when the new three‐compartment phenotyping concept was introduced recently [13]. The authors incorporated the patellofemoral compartment defined by the anterior trochlear angle in the transverse plane and reported a lack of correlation among parameters between the coronal and transversal plane. However, tracking the trochlear groove's orientation in the coronal plane may be more relevant, albeit more challenging to measure due to the three‐dimensional orientation shift from extension to flexion.

Knee position during capsule closure significantly affects patella mobility. In this study, all knees were positioned at 90° of flexion during closure to avoid restriction in patella mobility. The capsule is tight between 60° and 90° of knee flexion but is loose in extension. Clinical studies highlight that patella mobility after surgery is influenced by the knee position during capsule closure [25, 29]. Wound closure at 90° of knee flexion has been associated with less patella tilt and improved knee flexion and pain early after TKA compared to closure in extension.

Following the functional alignment principle, the femoral component is typically positioned in varus compared to conventional mechanical alignment due to the symmetrical design of the medial and lateral femoral condyles of the component. Given the variation in component placement, one might expect differences in patella mobility, such as reduced lateral patella mobility when the femoral component is placed in varus. The current study did not provide data about component placement in reference to Whiteside's line. Nonetheless, it has been demonstrated that functionally placed femoral components using a robotic platform allow for the most accurate trochlea depth restoration in all three planes [26].

Surgeon experience, intraoperative decision‐making and soft tissue balance likely also play prominent roles in patella mobility [22, 30]. Recent studies indicate that an increased offset of more than 5 mm may have clinical significance [18].

These findings align with recent studies showing no significant difference in functional outcomes and satisfaction scores between robotic and manual TKAs when the same prosthesis model is used [12, 22, 30].

Interestingly, the patellofemoral compartment seems to be more forgiving than expected, with recent findings indicating that a patella tilt of more than 5° did not impact clinical outcomes [8]. However, the boundaries remain unclear.

All measurements in the current study were taken in knee extension to eliminate the effect of the medial and lateral trochlear facet on the mediolateral patella shift. At 30° of flexion, the patella engages in the trochlear groove, guiding patella movement during further knee flexion [1].

A limitation of the current study is that patients were not randomized into separate groups. All surgeries were performed by three surgeons, each with over 15 years of experience in TKA surgery. Furthermore, no data about patella kinematics was recorded, in addition to providing information about knee function during motion.

## CONCLUSION

No significant difference in patella mobility was found between conventional mechanical aligned and RA functional aligned TKA. The extent of change in patella mobility did not correlate with clinical outcomes. Further research incorporating patella kinematics during knee motion and patient‐reported outcomes, and long‐term follow‐up is warranted.

## AUTHOR CONTRIBUTIONS

The authors have nothing to report.

## CONFLICT OF INTEREST STATEMENT

The authors declare no conflicts of interest.

## ETHICS STATEMENT

This study was approved by the University of Brandenburg, No. E‐02‐20200405.

## Data Availability

The authors have nothing to report.

## References

[1] Amis AA . Current concepts on anatomy and biomechanics of patellar stability. Sports Med Arthrosc. 2007;15:48–56. 10.1097/JSA.0b013e318053eb74 17505317

[2] Barink M , Van de Groes S , Verdonschot N , De Waal Malefijt M . The difference in trochlear orientation between the natural knee and current prosthetic knee designs; towards a truly physiological prosthetic groove orientation. J Biomech. 2006;39:1708–1715. 10.1016/j.jbiomech.2005.04.027 15979082

[3] Becker R , Karlsson J . The importance of the third knee compartment on outcome following total knee arthroplasty. Knee Surg Sports Traumatol Arthrosc. 2014;22:475–477. 10.1007/s00167-014-2860-5 24482214

[4] Colwell CW , Chen PC , D'Lima D . Extensor malalignment arising from femoral component malrotation in knee arthroplasty: effect of rotating‐bearing. Clin Biomech. 2011;26:52–57. 10.1016/j.clinbiomech.2010.08.009 20869142

[5] Crossley KM , Stefanik JJ , Selfe J , Collins NJ , Davis IS , Powers CM , et al. 2016 Patellofemoral pain consensus statement from the 4th International Patellofemoral Pain Research Retreat, Manchester. Part 1: terminology, definitions, clinical examination, natural history, patellofemoral osteoarthritis and patient‐reported outcome measures. Br J Sports Med. 2016;50:839–843. 10.1136/bjsports-2016-096384 PMC4975817 27343241

[6] Dahlmann S , Ziegeler K , Mau‐Möller A , Mittelmeier W , Bergschmidt P . Patellar tracking in total knee arthroplasty—influence on clinical and functional outcome. Diagnostics. 2022;12:1082. 10.3390/diagnostics12051082 PMC9139248 35626238

[7] Dai Y , Yin H , Xu C , Zhang H , Guo A , Diao N . Association of patellofemoral morphology and alignment with the radiographic severity of patellofemoral osteoarthritis. J Orthop Surg. 2021;16:548. 10.1186/s13018-021-02681-2 PMC8418017 34481492

[8] Diquattro E , Prill R , Salzmann M , Traina F , Becker R . High three‐dimensional accuracy of component placement and lower limb alignment using a robotic arm‐assisted system and gap‐balancing instrument in total knee arthroplasty. Knee Surg Sports Traumatol Arthrosc. 2024;32:685–692. 10.1002/ksa.12088 38415872

[9] Diquattro E , Andriollo L , Koutserimpas C , Baltzer J , Servien E , Batailler C , et al. Patellofemoral alignment safe zones in robotic‐assisted TKA do not affect outcomes but do influence patellar resurfacing rates. Knee Surg Sports Traumatol Arthrosc. 2025;33(12):4234–4243. 10.1002/ksa.12786 PMC12747612 40679230

[10] Du Z , Chen S , Yan M , Yue B , Wang Y . Differences between native and prosthetic knees in terms of cross‐sectional morphology of the femoral trochlea: a study based on three‐dimensional models and virtual total knee arthroplasty. BMC Musculoskelet Disord. 2017;18:166. 10.1186/s12891-017-1529-x PMC5397712 28427385

[11] Dye SF , Vaupel GL , Dye CC . Conscious neurosensory mapping of the internal structures of the human knee without intraarticular anesthesia. Am J Sports Med. 1998;26:773–777. 10.1177/03635465980260060601 9850777

[12] Hampp E , Chughtai M , Scholl L , Sodhi N , Bhowmik‐Stoker M , Jacofsky D , et al. Robotic‐arm assisted total knee arthroplasty demonstrated greater accuracy and precision to plan compared with manual techniques. J Knee Surg. 2019;32:239–250. 10.1055/s-0038-1641729 29715696

[13] Hess S , Chelli S , Leclercq V , Lustig S , Graichen H , Hirschmann MT . Three‐compartment phenotype concept of total knee arthroplasty alignment: mismatch between distal femoral, posterior femoral, and tibial joint lines. J Arthroplasty. 2025;40:2023–2034. 10.1016/j.arth.2025.02.015 40049560

[14] Hochreiter B , Hess S , Moser L , Hirschmann MT , Amsler F , Behrend H . Healthy knees have a highly variable patellofemoral alignment: a systematic review. Knee Surg Sports Traumatol Arthrosc. 2020;28:398–406. 10.1007/s00167-019-05587-z 31256215

[15] Hull ML , Simileysky A , Howell SM . Differences in trochlear morphology of a new femoral component designed for kinematic alignment from a mechanical alignment design. Bioengineering. 2024;11:62. 10.3390/bioengineering11010062 PMC10812931 38247939

[16] Iranpour F , Merican AM , Dandachli W , Amis AA , Cobb JP . The geometry of the trochlear groove. Clin Orthop Relat Res. 2010;468:782–788. 10.1007/s11999-009-1156-4 PMC2816780 19915941

[17] Koutserimpas C , Giovanoulis V , Saffarini M , Bonnin M , Hirschmann MT , Lustig S . The effects of over‐ and under‐stuffing the anterior knee compartment in primary TKA: a systematic review. Knee Surg Sports Traumatol Arthrosc. 2025;34(3):1002–1013. 10.1002/ksa.70033 40932242

[18] Koutserimpas C , Saffarini M , Bonnin M , Hirschmann MT , Lustig S . Optimizing the patellofemoral compartment in total knee arthroplasty: is it time for dynamic assessment? Knee Surg Sports Traumatol Arthrosc. 2025;33:387–392. 10.1002/ksa.12450 39224026

[19] Lachowski K , Hammermeister F , Halenz B , Lieckefett F , Götze T , Prill R , et al. The patellostabilometer: a new device for quantification of mediolateral patella displacement. Sensors. 2023;23:1274. 10.3390/s23031274 PMC9920919 36772314

[20] Lachowski K , Prill R , Salzmann M , Becker R . Inferior patellar mobility before and after knee arthroplasty: a comparison with healthy knees. Knee Surg Sports Traumatol Arthrosc. 2024;32:1531–1538. 10.1002/ksa.12159 38544470

[21] Laubach M , Hellmann JT , Dirrichs T , Gatz M , Quack V , Tingart M , et al. Anterior knee pain after total knee arthroplasty: a multifactorial analysis. J Orthop Surg. 2020;28:2309499020918947. 10.1177/2309499020918947 32338135

[22] Marchand RC , Sodhi N , Anis HK , Ehiorobo J , Newman JM , Taylor K , et al. One‐year patient outcomes for robotic‐arm‐assisted versus manual total knee arthroplasty. J Knee Surg. 2019;32:1063–1068. 10.1055/s-0039-1683977 30959549

[23] Matz J , Lanting BA , Howard JL . Understanding the patellofemoral joint in total knee arthroplasty. Can J Surg. 2019;62:57–65. 10.1503/cjs.001617 PMC6351265 30693747

[24] Orsi AD , Shatrov J , Plaskos C , Kreuzer S . Personalized alignment techniques better restore the native trochlear groove compared to systematic alignment techniques in total knee arthroplasty. Knee Surg Sports Traumatol Arthrosc. 2024;32:915–928. 10.1002/ksa.12104 38426570

[25] Plate JF , Seyler TM , Halvorson JJ , Santago AC , Lang JE . Non‐anatomical capsular closure of a standard parapatellar knee arthrotomy leads to patellar maltracking and decreased range of motion: a cadaver study. Knee Surg Sports Traumatol Arthrosc. 2014;22:543–549. 10.1007/s00167-013-2369-3 23334622

[26] Shatrov J , Coulin B , Batailler C , Servien E , Walter B , Lustig S . Alignment philosophy influences trochlea recreation in total knee arthroplasty: a comparative study using image‑based robotic technology. Int Orthop. 2023;47:329–341. 10.1007/s00264-022-05570-3 PMC9877070 36112197

[27] Shervin D . Anterior knee pain following primary total knee arthroplasty. World J Orthop. 2015;6:795–803. 10.5312/wjo.v6.i10.795 PMC4644867 26601061

[28] Stoddard JE , Deehan DJ , Bull AMJ , McCaskie AW , Amis AA . No difference in patellar tracking between symmetrical and asymmetrical femoral component designs in TKA. Knee Surg Sports Traumatol Arthrosc. 2014;22:534–542. 10.1007/s00167-013-2534-8 23728417

[29] Wang S , Xia J , Wei Y , Wu J , Huang G . Effect of the knee position during wound closure after total knee arthroplasty on early knee function recovery. J Orthop Surg. 2014;9:79. 10.1186/s13018-014-0079-2 PMC4237872 25149657

[30] Wang Y , Lu J , Wang Z , Li Z , Pan F , Zhang M , et al. The association between patella alignment and morphology and knee osteoarthritis. J Orthop Surg. 2024;19:509. 10.1186/s13018-024-05001-6 PMC11348726 39192379

